# Efficacy of Pro-Kinetic Agents in Type 2 Diabetes Mellitus Patients With Gastroparesis Using Lactulose Hydrogen Breath Testing: A Randomized Trial

**DOI:** 10.7759/cureus.20990

**Published:** 2022-01-06

**Authors:** Prakash Tendulkar, Ravi Kant, Satyavati Rana, Poonam Yadav, Anissa A Mirza, Disha Agarwal

**Affiliations:** 1 Internal Medicine, All India Institute of Medical Sciences, Rishikesh, IND; 2 Biochemistry, All India Institute of Medical Sciences, Rishikesh, IND; 3 Nursing, All India Institute of Medical Sciences, Rishikesh, IND; 4 Public Health, All India Institute of Medical Sciences, Rishikesh, IND

**Keywords:** gcsi, octt, breath test, gastroparesis, diabetes mellitus

## Abstract

Aim

The aim of the study was to determine the efficacy of prokinetic agents in diabetic gastroparesis patients.

Method

This was a randomized open-label trial conducted on 50 patients with type 2 diabetes experiencing diabetic gastroparesis, which was diagnosed with the lactulose hydrogen breath test. After randomization, all 50 patients were divided into four arms (cinitapride, metoclopramide, levosulpiride, and domperidone) of different prokinetics and followed up for four weeks; after which, repeat gastroparesis cardinal symptom index score and orocecal transit time were recorded in order to assess the response to the treatment.

Result

There was no statistically significant difference among the four groups in terms of all the baseline characteristics except for gender (p=0.032). The follow-up gastroparesis cardinal symptom index was collected for 50 patients but repeat orocecal transit time could be performed only in 37 patients. In all four groups, there was a statistically significant (p<0.05) improvement in terms of orocecal transit time and gastroparesis cardinal symptom index scores. But there was no statistically significant difference in relative efficacy amongst these study groups.

Conclusion

Our study showed statistically significant improvement with four prokinetics drugs in terms of gastroparesis cardinal symptom index score and orocecal transit time, but there was no statistically significant benefit of one prokinetic drug over the other. Our study showed promising results with regard to prokinetic use in diabetic gastroparesis.

## Introduction

Diabetes-related autonomic neuropathy is a very common complication of long-standing diabetes mellitus which can involve multiple systems like cardiovascular, gastrointestinal, genitourinary, sudomotor, and metabolic systems. The most common and important manifestation of gastrointestinal autonomic neuropathy is gastroparesis [1]. "Gastroparesis is defined as delayed gastric emptying with associated symptoms in the absence of mechanical obstruction" [2].

The cardinal symptoms of gastroparesis are nausea, vomiting, abdominal bloating, early satiety, upper abdominal pain. Patients can have other severe complications like weight loss, malnutrition, dehydration, electrolyte imbalance, bezoar formation, and aspiration pneumonia. There is significant overlap between gastroparesis and other upper gastrointestinal disorder, such as functional dyspepsia which makes the diagnosis even more challenging. There are various causes of gastroparesis like diabetes mellitus, hypo/hyperthyroidism, post-viral, autoimmune diseases, secondary to medications - opioids, tricyclic antidepressants, beta-blockers, calcium channel blockers, and post-surgical effects. Pathogenic mechanisms of both upper and lower gastrointestinal dysfunction include abnormalities of motor function, visceral hypersensitivity, altered secretion of GI hormones, inflammatory state, autonomic dysfunction, and genetic predisposition [3]. Diabetes leads to an imbalance between inhibitory and excitatory enteric neuropeptide ratios, which can directly cause altered gut motility. A reduction in insulin/insulin growth factor 1 (IGF-I) signaling in diabetes leads to interstitial cells of Cajal (ICC) depletion, subsequent stem cell factor depletion, and resultant smooth muscle atrophy. In this context, insulin is of paramount importance. In diabetes, IGF-I is reduced, resulting in smooth muscle atrophy, which, in turn, contributes to impaired gastrointestinal motility [4].

Diabetes affects gastric motor function more than small bowel transit, indicating an increased sensitivity of the stomach to diabetic injury. Therefore, a confirmed diagnosis of gastroparesis requires measurement of delayed gastric emptying via an appropriate test. Diabetic patients who are having cardinal symptoms of gastroparesis can undergo delayed gastric emptying tests like gastric emptying scintigraphy (GES), breath test with stable isotopes, wireless motility capsule (WMC), radiopaque marker, MRI, single-photon emission computed tomography, electrogastrography, etc. The diagnosis of gastroparesis is usually made on the basis of a combination of symptoms suggestive of gastroparesis and objective evidence of delayed gastric emptying after ruling out gastric outlet obstruction. Objective assessment of delayed gastric emptying is the mandatory criteria for making a definite diagnosis of gastroparesis [3,5]. 

Gastric emptying scintigraphy (GES) is the most widely available and gold standard procedure for the measurement of delayed gastric emptying [5]. The American Neurogastroenterology and Motility Society recommend the use of a 99 mTc (Technetium) sulfur colloid-labeled egg, two slices of bread, strawberry jam, and water (255 Kcal) for diagnosis of gastroparesis [6,7]. The initial management of gastroparesis starts with the restoration of fluids/electrolytes, nutritional supplementation, and in diabetics, optimization of glycaemic control [8,9].

This technique, although considered to be the gold standard, fails to identify early cases; also, the study is related to the stomach part of the gastrointestinal system, whereas the dysmotility changes take place in the entire small intestine. It is thus the need of the hour to exploit a test that can address the entire gut and especially those cases with normal GES [5].

Hydrogen breath tests are widely used to explore the pathophysiology of functional gastrointestinal disorders. Small intestinal bacterial overgrowth (SIBO) and carbohydrate malabsorption are the main disorders detected by these tests that have been proposed to be of great importance for symptoms of gastrointestinal (GI) diseases. Although there are various types of breath tests yet glucose hydrogen breath test is more acceptable for the diagnosis of SIBO and lactose, fructose, sucrose hydrogen breath tests are used for detection of lactose, fructose, and sucrose malabsorption respectively. Lactulose hydrogen breath test is used widely to measure orocecal transit time (OCTT) for GI motility [10].

These methods are non-invasive, inexpensive, and simple and can address the gut motility of the entire small intestine and not restricted to the stomach as in the case of GES, as there is a sizeable number of cases that are reported to be abnormal on GES despite having symptoms of gastroparesis. As this is a non-invasive method, it can be exploited not only to see the gastroparesis but to assess the pharmacological effects of drugs used in the treatment of diabetic gastroparesis. Therefore we conducted this randomized control study to see and compare the efficacy of prokinetic agents used in the treatment of diabetic gastroparesis [10].

## Materials and methods

Objectives

The primary objective was to determine the efficacy of prokinetic agents in diabetic patients with diabetic gastroparesis and the secondary objective was to compare the relative efficacy between the prokinetic agents in diabetic gastroparesis patients.

Study design

It was an open-label randomized trial.

Study setting

Diabetic clinic in the outpatient department (OPD) of internal medicine of a tertiary care teaching hospital in North India.

Study duration

The study was conducted over a period of 18 months from January 2020 to July 2021.

Patient selection

Inclusion criteria were type 2 DM patients aged 18-65 years (males and female) known/diagnosed with delayed OCTT on lactulose hydrogen breath test; the exclusion criteria were all type 2 diabetic patients in whom gastroparesis was caused by drug intake like narcotic pain medicines, antidepressants, anticholinergics, amylin analogues, those who underwent vagotomy, gastric surgery, had thyroid disorder (hypothyroidism), Parkinsonism or who were pregnant.

Study sample size

A consecutive sampling method was used to enroll all the eligible patients from January 2020 to September 2020 for this pilot project. All eligible patients with type 2 diabetes mellitus and with delayed OCTT were included in the study during the study period. in view of the COVID-19 pandemic, restrictions in the form of countrywide lockdown from 24th March 2020 onwards, led to the enrollment of 50 patients [11]. These patients were further randomly assigned to four groups.

Randomization

All patients fulfilling the inclusion criteria were randomly assigned to four groups. The sequence generation was done by simple random allocation using a computer-based software program QuickCalcs.

Intervention

**Figure 1:**
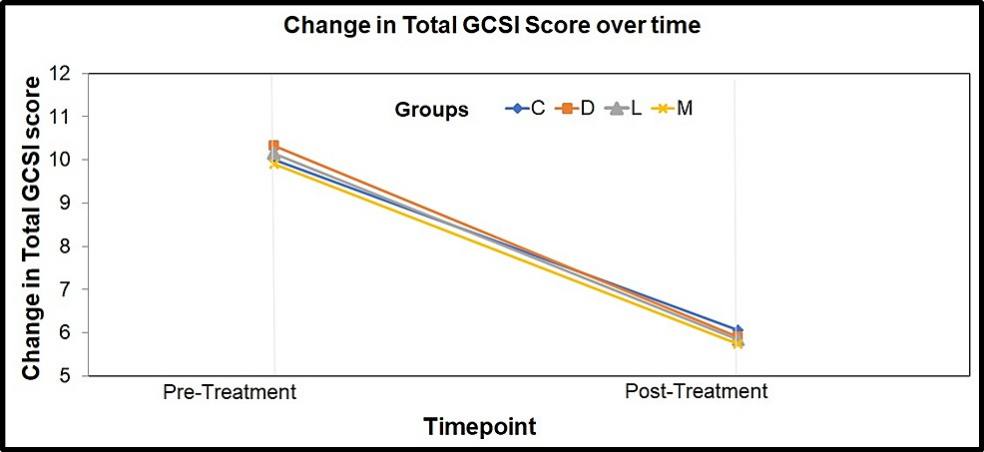
Line diagram depicting the change in the mean pre- and post-GCSI scores of the four intervention groups GCSI - gastroparesis cardinal symptoms index; C - cinitapride; D - domperidone; L - levosulpiride; M - metoclopramide

Patients who presented with at least two gastrointestinal symptoms like nausea, vomiting, bloating/distension, or early satiety were assessed by gastrointestinal cardinal symptoms index. The initial score of these patients was noted, they were then subjected to lactulose hydrogen breath test those who had delayed gastric emptying by lactulose hydrogen breath test were included in the study (Figure 1). They were randomly allocated to four groups regarding group 1 cinitapride (group ‘C’) group 2 levosulpiride (group ‘L’) group 3 and 4 received metoclopramide and domperidone and hence termed as group ‘M’ and group ‘D’ respectively. The starting therapeutic dosage of these drugs were 1 mg thrice daily for cinitapride, 75 mg once daily for levosulpiride, 10 mg thrice daily for metoclopramide, and domperidone. The treatment so instituted was continued for four weeks following which these patients were reassessed by GCSI and lactulose hydrogen breath test and the pre and post-intervention scores were assessed and subsequently analysed.

Data entry and analysis 

The data was entered in MS Excel 2019 for coding and checking for any missing values or duplications of entries. Categorical variables are reported as percentages and continuous or discrete variables are reported as means ± standard deviation (SD). The paired Student t-test for continuous variables and the ANOVA test were used for comparisons between the groups. Data were presented as odds ratios (OR) with 95% confidence intervals (CI). SPSS Statistics v. 26.0 (IBM Corp., Armonk, NY) was used for statistical analysis and statistical significance was set at p < 0.05 for the final analysis.

Ethical considerations

The study was conducted after getting approval from the ethics committee of AIIIMS Rishikesh (Ref no. AIIMS/IEC/20/802) and the trial was registered with the Clinical Trial Registry of India (CTRI) under the registration number CTRI/2020/05/033594.

Confidentiality of data

Confidentiality of the information obtained about the patients was maintained and the identity of the patient was not revealed. Data, if shared, was anonymised.

## Results

A total of 50 study participants were included in the study. The baseline demographic profile is shown in Table 1. Out of these 50 participants, 31 (62%) were male and 19 (38%) were females. They were divided into four groups [(metoclopramide(M), levosulpiride (L), domperidone (D), cinitapride (C)] and there was a statistically significant difference noted between the study participants in terms of gender distribution (p-value= 0.032). 

**Table 1:** Baseline characteristics of the study participants according to the intervention arms M - metoclopramide; L - levosulpiride; D - domperidone; C- cinitapride; TLC - total leucocyte count; FBS - fasting blood sugar; PPBS - post-prandial blood sugar; TSH - thyroid-stimulating hormone; FT4 - free T4

Variables	Intervention Groups					
	M (N=12)	L (N=13)	D (N=12)	C (N=13)	p-value	
Gender						
Male	6 (50%)	10 (77%)	4 (33%)	11 (85%)	0.032	
Female	6 (50%)	3 (23%)	8 (67%)	2 (15%)		
Age (years)	54.4±7.23	54±9.1	56.75±11.5	51.23±10.35	0.158	
Height (cm)	160.33±7.05	166.61±10.51	157.58 ±5.23	164.77±3	0.240	
Weight (Kg)	64.25±8.65	68.69±9.9	63.33±7.27	71.92±10.75	0.182	
BMI (Kg/m^2^)	24.96±2.39	24.7±2.03	25.79±3.98	26.5±3.77	0.05	
18.5-22.9 kg/m^2^	3 (25.0%)	1 (7.69%)	3 (25.0%)	2 (15.38%)		
23.0-24.9 kg/m^2^	4 (33.33%)	7 (53.85%)	2 (16.67%)	4 (30.77%)		
≥25.0 kg/m^2^	5 (41.67%)	5 (38.46%)	7 (58.33%)	7 (53.85%)		
Haemoglobin (g/dl)	12.45±1.53	13.28±1.65	12.16±1.18	13.13±1.07	0.335	
TLC (per mm^3^)	6.64±1.69	7.61±1.09	6.48±1.1	7.22±0.98	0.816	
Urea (mg/dl)	46.58±12.71	43.07±25.16	42.25±15.44	36.84±12.83	0.901	
Creatinine (mg/dl)	1.09±0.29	1.13±0.76	1.08±0.43	0.85±0.31	0.104	
≤1.1 mg/dl	7 (58.33%)	9 (69.23%)	8 (66.67%)	12 (92.31%)		
>1.1 mg/dl	5 (41.67%)	4 (30.77%)	4 (33.33%)	1 (7.69%)		
FBS (mg/dl)	212.41±48.07	220.46±45.07	238.92±63.47	205.92±27.99	0.197	
PPBS (mg/dl)	296.16±76.06	294.3±52.35	313.08±71.2	272.7±43.43	0.965	
HbA1c (%)	9.58±2.73	9.3±1.54	9.5±1.72	8.48±0.68	0.821	
6-8%	3 (25.0%)	3 (23.08%)	4 (33.33%)	2 (15.38%)		
8.1-10%	6 (50.0%)	7 (53.84%)	5 (41.67%)	11 (84.62%)		
>10%	3 (25.0%)	3 (23.08%)	3 (25.0%)	0		
TSH (mIU/L)	3.13±0.65	3.4±0.72	3.42±0.38	3.4±0.75	0.957	
FT4 (ng/dl)	1.13±0.11	1.25±0.2	1.18±0.11	1.12±0.13	0.830	
Duration of diabetes (years)	7.04±3.14	6.8±4.77	6.92±5.37	7±4.37	0.637	

There was no statistically significant difference among the four groups in terms of all the baseline characteristics except for gender (p=0.032), taking a significant p-value of <0.05.

Our study showed a statistically significant difference between the mean pre and post GCSI scores (p<0.001) along with a significant reduction in their mean pre and post-scores. Relative change in GCSI score in four arms is shown in Figure 1.

**Figure 2:**
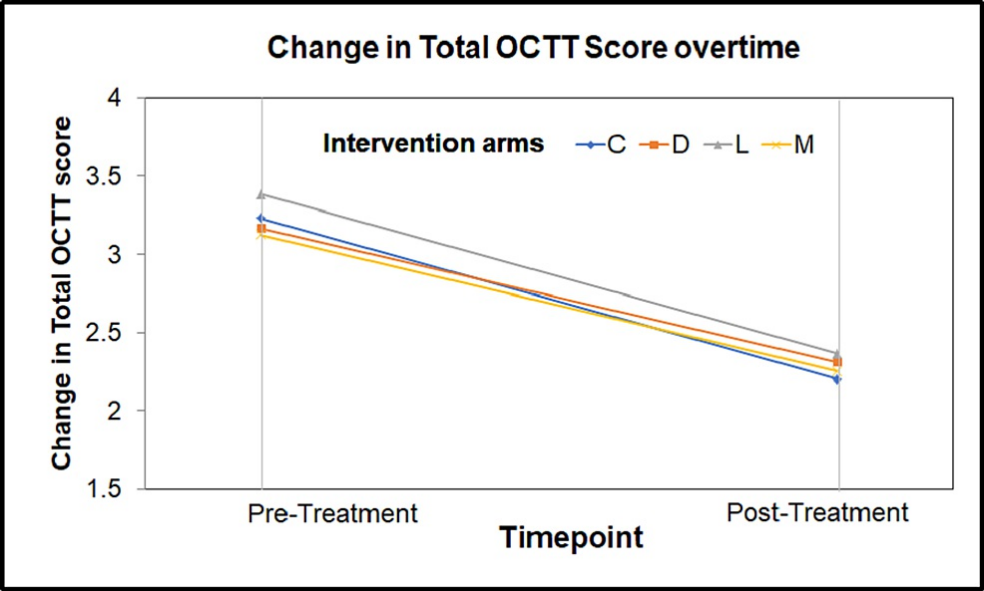
Line diagram depicting the change in the mean pre- and post-OCTT (in hours) of the four intervention groups OCTT- orocecal transit time; C - cinitapride; D - domperidone; L - levosulpiride; M - metoclopramide

Out of 50 patients enrolled in the trial, post-intervention OCTT was recorded only for 37 study participants. There was a statistically significant difference between the mean pre and post OCTT scores (p<0.001 for cinitapride and levosulpiride arms, p=0.002 in the domperidone arm and p=0.003 in the metoclopramide arm), along with a significant reduction in their mean pre- and-post OCTT scores. Relative change in OCTT in four arms is shown in Figure 2.

We also compared different prokinetics with metoclopramide (the only FDA-approved drug), we found no statistically significant difference in the mean differences (post-GCSI scores to pre GCSI scores) of the three prokinetic drugs (cinitapride, domperidone, and levosulpiride) when compared to that of metoclopramide. (p=0.666 for arm C, p=0.657 for arm D and p=0.814 for arm L) when compared with metoclopramide)

We also found no statistically significant difference in the mean differences (post-OCTT scores to pre OCTT scores) of the three prokinetic drugs (cinitapride, domperidone, and levosulpiride) when compared to that of metoclopramide. (p=0.465 for arm C, p=1.0 for arm D and p=0.703, when compared with metoclopramide).

Table 2 represents a comparison among the four prokinetic drugs to check for the efficacy of these drugs in terms of the GCSI scores. The maximum reduction in the mean GCSI scores was seen in the domperidone group, but there was no statistically significant difference in the four groups (p=0.815). this table also represents a comparison among the four prokinetic drugs to check for efficacy of the drugs in terms of the OCTT scores, there was no statistically significant difference in the four groups so far as OCTT is concerned (p=0.843).

**Table 2:** Comparison of the efficacy of the four prokinetic drugs among themselves based on their GCSI score and OCTT (in hours) GCSI - gastroparesis cardinal symptom index; OCTT- orocecal transit time

Intervention arms	n (GCSI)	Mean Diff±SD (GCSI scores)	p-value	n (OCTT)	Mean Diff±SD (OCTT- in Hours)	p-value
Cinitapride	13	-3.92±1.25	0.815	10	-1±0.53	0.843
Domperidone	12	-4.42±1.17		8	-0.81±0.46	
Levosulpiride	13	-4.31±1.44		11	-0.91±0.54	
Metoclopramide	12	-4.17±1.53		8	-0.81±0.53	

## Discussion

This study has clearly shown that the four prokinetic agents, i.e. levosulpiride, metoclopramide, domperidone, and cinitapride are equally effective in diabetic gastroparesis patients in alleviating the symptoms as well as reducing the orocecal transit time measured by a hydrogen breath test.

Although there was a statistically significant difference between the two sexes, study groups were well matched as far as age, height, weight, BMI, and mean duration of diabetes mellitus. These groups were also similar in terms of laboratory parameters like FBS, PPBS, and HbA1c, urea, and creatinine.

There are a few studies that utilized the GCSI as a basis to analyze the response of treatment to prokinetic drugs. One of the landmark studies was done by Heckert and Parkman, wherein they documented the effects of domperidone on GCSI score in patients who had gastric emptying scintigraphy proven gastroparesis; although the study included diabetic and non-diabetic patients, there was a major improvement in the symptom score of these patients. The idea behind the study was to compare the utility of gastroparesis cardinal symptom index by maintaining a daily diary (GCSI-DD) and compare the score with other assessment tools like patient assessment of upper gastrointestinal symptom index (PAGI-SYM) that incorporates GCSI and clinical patient grading assessment scale (CPGAS). The researchers could document very clearly that the GCSI-DD was as good as PAGI-SYS in not only identifying patients with gastroparesis but the response to domperidone as well. There was a strong correlation amongst these two scales and strong agreement was seen in symptoms resolution at two weeks’ time from baseline (r = 0.49, P=0.004). These findings make GCSI score a valid tool for assessing the improvement of prokinetic agent domperidone and presumably to other available agents in this category. This study documented a significant improvement in nausea, early satiety, and postprandial fullness as well. It also concluded that GCSI is as good a marker of not only in assessing the severity of gastroparesis as PAGI-SYS but in assessing the response to treatment as well [12].

In our study, we found that the mean GCSI in the pre-treatment phase was comparable in all the groups ranging from 9.92±2.53 in metoclopramide to 10.3±1.61 in the domperidone group. All groups showed a remarkable reduction in the GCSI score with a highly significant p-value (< 0.001) in the post-treatment phase when compared to the pre-treatment period. These findings are in concurrence with what was observed by Heckert and Parkman [12]. This also suggests that prokinetic drugs have almost equal efficacy in reducing the GCSI across groups Although found to be equally efficacious by GCSI, the effect needs to be assessed more objectively as the GCSI is more or less a subjective score. In order to assess the efficacy of these drugs in a more objective manner either gastric scintigraphy studies, wireless motility capsule, or OCTT is used. The latter was used in this study. There is a paucity of data on OCTT using lactulose hydrogen breath test for assessing the gastric motility in diabetic subjects in general and the response to treatment in particular.

The role of OCTT was convincingly assessed by Burge and colleagues who used the standardized potato meal with lactulose and assessed the diabetic patients with gastroparesis, diabetic patients without gastroparesis, and non-diabetic subjects and followed them with H2 breath testing every 30 mins for 24 hours. All these patients were diagnosed on the basis of gastric emptying scintigraphy and were then classified into the aforementioned groups. The researchers found that the group with diabetic gastroparesis had a prolonged H2 breath test as compared to those diabetic patients who didn’t have gastroparesis vis-à-vis non-diabetic. This study concluded that OCTT can be reliably used as a marker to diagnose not only diabetic gastroparesis with confidence but small intestinal dysmotility as well [13].

The OCTT has a wide variability of normal transit time; the Rome consensus conference (2009) specified that normal OCTT ranges between 40- and 170-min for lactulose meal and between 192 and 232 min for a solid meal [14]. Minocha et al. (1995) stated that OCTT in an average healthy population was 103.2±11.2 min. They conducted the study to see the role of erythromycin in diabetic gastroparesis [15]. Studies conducted in the north Indian population also showed variation in normal OCTT. Rana et al., in four different studies, showed OCTT ranging between 90.6 ± 10.4 min to117±6 min [10]. So we took a cut of more than two hours (>120min) as delayed OCTT in our patients. In our study, minimum pre-intervention OCTT was seen in the metoclopramide group. In contrast, minimum post-intervention OCTT was seen in the cinitapride group. However, the average time for the lactulose-based hydrogen breath test is 90 mins using 10 grams of lactulose; an amount more than this increases gastric motility. Transit is relatively slower in healthy elderly.

Metoclopramide is considered to be the drug of choice in diabetic gastroparesis; the drug had been endorsed by USFDA for its use in this clinical condition based on the earlier control trials using gastric emptying studies to prove its efficacy [16]. This drug remained the mainstay of therapy until recent times as safer options are available to these patients. The adverse effects associated with metoclopramide are among the main concerns for its chronic use. Neurological side effects like anxiety, depression, somnolence, and headache are the main adverse events associated with this drug [17].

The readily available prokinetic drugs like levosulpiride, cinitapride, and domperidone merit prescription but lack scientific evidence of their clinical efficacy. Domperidone was found to be effective in diabetic gastroparesis patients at a dose of 10 mg thrice daily. Patterson et al. also showed it to be better than metoclopramide especially with regard to central adverse effects [18]. Another study (DOM-USA-5 study group, 1998) during the same time showed that domperidone 20mg QID significantly improved the gastroparesis symptoms and was well tolerated in diabetic patients [19]. Even some studies and meta-analyses showed domperidone to be more superior to metoclopramide in alleviating the gastroparesis symptoms in diabetic patients [20]. In line with the previous finding, our study depicted statically significant improvement in post-intervention GCSI score from 10.3±1.61 to 5.92±1.37 (p<0.001) and statically significant improvement in post-intervention OCTT from 3.12±0.58 hrs (187±35 min) to 2.31±0.26 hrs (139±16 min) (p< 0.002)

Levosulpiride is an antidopaminergic drug that improves gastric motility. Mansi et al. stated that levosulpiride is equally efficacious to cisapride in shortening the t½ of gastric emptying and improving the total symptoms score. This study also stated that levosulpiride effectively improved the symptom impact on the patients' daily activity and improved symptoms like nausea, vomiting, and early post-prandial satiety. The study sample size was only 30 patients, and they used a 13C-octanoic acid breath test to document delayed emptying [21]. In another study, Melga et al. showed that in insulin-dependent diabetes mellitus (IDDM) patients, levosulpiride significantly reduced gastric emptying (p < 0.001) [22]. Our study also showed statically significant (p <0.001) improvement in the pre- to post-intervention GCSI score from 10.15±2.51 to 5.85±1.62. Also, there was statically significant (p <0.001) pre and post-intervention OCTT from 3.27±0.68 hr (196±41 min) to 2.36±0.32 (142±19 min). Compared to levosulpiride to metoclopramide, there was no statistically significant difference between the two drugs in terms of pre-intervention and post-intervention GCSI scores and OCTT difference. (p= 0.814 and p=0.703 respectively).

Cinitapride is a 5-HT4 receptor agonist and leads to increased gastric motility. One of the RCTs conducted in Italy with patients with dysmotility like dyspepsia and delayed gastric emptying with placebo showed no statistically significant difference (ANOVA, p=0.8720) in improving gastric emptying. However, the decrease was greater for the cinitapride group (ANOVA, p=0.0169) [23]. Our study showed statistically significant (p <0.001) improvement in pre- and post-intervention GCSI scores from 10.0±2.08 to 6.08±1.55. Also, there was statically significant (p <0.001) pre and post-intervention OCTT from 3.20±0.63 hr (192±40min) to 2.20±0.35 (132±21 min). Comparing cinitapride to metoclopramide, in terms of pre-intervention and post-intervention GCSI scores and OCTT, there was no statistically significant difference between the two drugs (p= 0.666 and p=0.465 respectively). Our study showed significant improvement with all the drugs in terms of GCSI score and OCTT, but no statically significant benefit of one prokinetic drug over the other in terms of GCSI score and OCTT.

The treatment of gastroparesis in diabetic patients portends a better glycaemic control also, Melga et al. demonstrated a better glycaemic control in terms of reduction in HbA1c in levosulpiride treated group against placebo. They found no statistically significant reduction in HbA1c in the placebo-treated group; the levosulpiride group on the other hand showed a significant reduction (HbA1c 6.7±0.4% and 5.7±0.3%, P <0.01) at the start and end of the study. The study was done on patients receiving insulin therapy and with diabetic gastroparesis. These patients showed a remarkable reduction in gastric emptying time after six months of levosulpiride use [22].

Our study also had few limitations as much of the data collection period was obstructed by the COVID-19 pandemic leading to multiple nationwide lockdowns. Due to the pandemic, the footfall of regular patients had fallen drastically, especially impacting our study on stable diabetic patients. The study was done only for four weeks, so long-term side effects were not studied, but no severe side effects were documented within that time. Also, no blinding was used in the study. Another limitation to the study was the absence of any post hoc analyses.

Although there are many studies showings the beneficial effects of prokinetic agents in the treatment of diabetic gastroparesis using different agents in various clinical settings, there is however lack of head-to-head trial amongst these drugs and hence there is paucity in the superiority of one over other. There are issues related to cost as well. The OCTT has been sparingly used in the past for at least assessing the response of various prokinetic drugs in diabetic gastroparesis. The studies by Rana et al. have documented the normal gastric emptying time in normal as well as in diabetic Indian patients [10]. The findings in the current study suggest an improvement in the transit time by the use of prokinetic agents. These drugs are comparable and have equal potential in alleviating the symptoms of nausea, early satiety, and abdominal bloating.

## Conclusions

To the best of our knowledge, this is the first study comparing the relative efficacy of levosulpiride, cinitapride, metoclopramide, and domperidone. Our study showed significant improvement with four prokinetics drugs in terms of gastroparesis cardinal symptom index score and orocecal transit time, but there was no statistically significant benefit of one prokinetic drug over the another seen in terms of GCSI score and OCTT. Although the sample size was small as the study was conducted during the COVID-19 pandemic, our study nevertheless showed promising results with regard to prokinetic use in diabetic gastroparesis. Also, our study showed very positive role of lactulose hydrogen breath test (LHBT) in the diagnosis and follow-up of the patients. Moreover, these results call for larger multicenter trials in order to derive more conclusive and promising alternatives for gastroparesis in the future.
